# Mullerian adenosarcoma of the uterus associated with tamoxifen treatment for breast cancer

**DOI:** 10.4274/tjod.44341

**Published:** 2015-12-15

**Authors:** Yasin Ceylan, Emek Doğer, Ahmet Yiğit Çakıroğlu, Çiğdem Vural, İzzet Yücesoy

**Affiliations:** 1 Kocaeli University Faculty of Medicine, Department of Obstetrics and Gynecology Kocaeli, Turkey; 2 Kocaeli University Faculty of Medicine, Department of Pathology, Kocaeli, Turkey

**Keywords:** Breast cancer, tamoxifen, uterine Mullerian adenosarcoma

## Abstract

Mullerian adenosarcoma following tamoxifen therapy is a rare condition. Our aim was to report the youngest patient in the literature with uterine mullerian adenosarcoma who was undergoing tamoxifen therapy for breast cancer. A premenopausal woman aged 38 years who was undergoing tamoxifen therapy for breast cancer, was admitted with symptoms of lower abdominal pain and irregular vaginal bleeding and malodorous vaginal discharge that had continued for at least 6 months. A pelvic examination revealed a large and malodorous polypoid mass protruding through the cervix and an enlarged uterus. A biopsy from the protruding polypoid mass was reported as a large area of necrosis with neoplastic mesenchymal cells. The patient underwent a total abdominal hysterectomy, bilateral salpingo-oopherectomy, pelvic-paraaortic lymph node dissection, and omentectomie. The histologic diagnosis was Mullerian adenosarcoma. As a result, she was discharged to the oncology department. The woman is alive and her chemoradiotherapy treatment is ongoing.

The role of tamoxifen therapy in the development of endometrial neoplasms remains unclear, but all cases of endometrial thickening and vaginal bleeding must be investigated for Mullerian adenosarcoma in tamoxifen users.

## INTRODUCTION

Mullerian adenosarcoma is an uncommon variant of mullerian mixed tumor of the uterus. Mullerian adenosarcoma accounts for only about 5-8% of all uterine sarcomas and more seldom on extrauterine sites including the cervix, ovary, vagina, fallopian tubes, and intestinal serosa. There have been a number of case reports of mullerian adenosarcoma arising in women undergoing tamoxifen or toremifene treatment or with endogenous hyperestrogenism^(1)^.

Tamoxifen has been shown to increase survival for women with breast cancer and decrease the risk of estrogen receptor-positive breast cancer in high-risk female populations^(2)^. Despite its benefits, tamoxifen has weakly estrogenic effects that can produce endometrial cell proliferation and increases the risk of endometrial cancer. However, the magnitude of increased risk remains unclear, recent studies indicate that the risk associated with tamoxifen may be substantially higher for rare, aggressive forms of uterine tumors, notably uterine sarcomas^(2,3,4,5,6,7,8)^. To date, 16 cases of mullerian adenosarcoma of the uterus associated with tamoxifen therapy have been reported in the English literature^(2,3,4,5,6,7,8,9,10,11)^.

Although most patients were diagnosed between the age of 40-65 years, 10% were aged less than 40 years^(1)^. In the present study, we report the youngest woman in the literature with uterine mullerian adenosarcoma who was undergoing tamoxifen therapy for breast cancer.

## CASE REPORT

A multiparous woman aged 38 years was admitted to our hospital with symptoms of lower abdominal pain and irregular vaginal bleeding and malodorous vaginal discharge that had continued for at least 6 months. She had a history of breast cancer. She had undergone a modified radical left mastectomy including left axillary dissection four years ago. A pathologic examination had revealed a grade 3, progesterone receptor-positive and c-erb-B2 score 3, but estrogen receptor-negative invasive ductal carcinoma. Post-operative treatment consisted of four chemotherapy courses using the CAF regimen (cyclophoshamide 600 mg/m^2^, adriamycin 60 mg/m^2^, 5-FU 600 mg/m^2^) and nine weeks of herceptin 6 mg/kg and docetaxel 75 mg/m^2^. After chemotherapy courses, she had been treated with 20 mg/day tamoxifen (Nolvadex, AztraZeneca Inc, UK) for 30 months.

**Figure 1:**
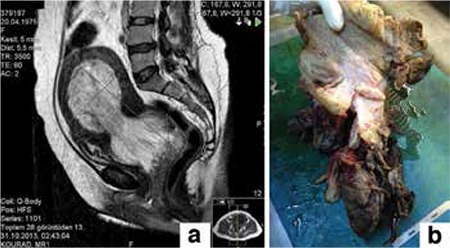
a: Magnetic resonance images showed a bilobular solid heterogeneous mass that filled the endometrial cavity. b: Macroscopically, the tumor was polipoid lesion extending into the endometrial cavity

**Figure 2:**
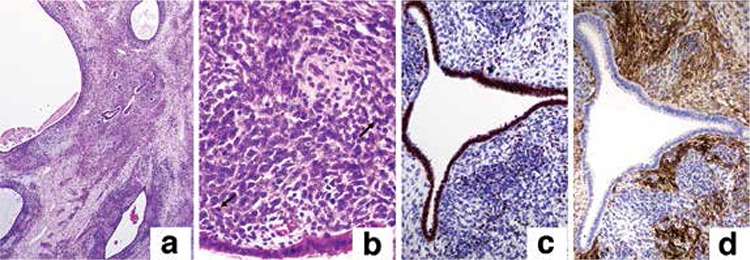
a: The tubular glands were lined by a benign proliferative endometrioid-type epithelia and the mesenchymal component consisted of low-grade sarcoma, which was typically more cellular around the glands resulting in periglandular cuffs. b: The sarcomatous cells appeared to contain focal atypia and pleomorphic endometrial stromal cells or fibroblasts. c: Immunohistochemically, the benign-appearing epithelial cells and sarcomatous tumor cells showed nuclear reaction with progesterone receptor and estrogen receptor d: Immunohistochemically, stromal cells showed cytoplasmic reaction with CD10

On admission, a pelvic examination revealed a large and malodorous polypoid mass protruding through the cervix and an enlarged uterus. Magnetic resonance images showed that a 192x100 mm uterus and a 75x65 mm solid heterogeneous mass filled the endometrial cavity without signs of invasion to surrounding tissues (Figure 1a). A biopsy from the protruding polypoid mass was reported as a large area of necrosis with neoplastic mesenchymal cells. Fresh frozen sections taken during the operation revealed an endometrial stromal sarcoma so a total abdominal hysterectomy, and bilateral salpingo-oopherectomy were performed. Pelvic-paraaortic lymph node dissection and omentectomy were added because there were palpable lymph nodes. Macroscopically, the tumor appeared as a polipoid lesion that extended into the endometrial cavity (Figure 1b). The cut surface of tumor showed cystic spaces filled with watery or mucoid fluid, surrounded by tan to gray tissue. More than half of the tumor was myometrial invasion. Histopathologically, the tumor was a biphasic lesion with benign epithelial elements and a sarcomatous stroma. Tubular glands, cysts, and cleft-like spaces were distributed throughout the tumor. These glands were lined by a benign proliferative endometrioid type epithelia. The mesenchymal component consisted of low-grade sarcoma, which was typically more cellular around the glands resulting in periglandular cuffs (Figure 2a). In these cellular areas, there was a maximum of 9 mitotic figures/10 HPF; the sarcomatous cells appeared to contain focal atypia and pleomorphic endometrial stromal cells or fibroblasts (Figure 2b). Immunohistochemically, the benign-appearing epithelial cells and sarcomatous tumor cells showed nuclear reaction with progesterone receptor and estrogen receptor (Figure 2c), and stromal cells showed cytoplasmic reaction with CD10 (Figure 2d). The final histologic diagnosis was mullerian adenosarcoma; the cervix, bilateral adnexa, pelvic-paraaortic lymph nodes, and omentum appeared unremarkable. The woman had stage IC disease according to the 2009 International Federation of Gynecology and Obstetrics Staging System for Uterine Adenosarcoma.

As a result, the patient was treated with adjuvant chemotherapy courses using doxorubicin, mesna, and ifosfamide (Day 1: Doxorubicin 50mg/m^2^ over 15 minutes, followed by ifosfamide 5g/m2 via 24-hour continuous IV admixed with mesna 6g/m2 for 36-hour through continuous IV. The cycle is being repeated every 3 weeks). After four chemotherapy courses, she is still alive although with vertebral bone metastasis.

## DISCUSSION

Tamoxifen has been used for the management of breast cancer for more than 40 years^(7)^. Despite its therapeutic effect on breast carcinoma, its estrogenic effect on the endometrium may also be complicated by a number of endometrial lesions such as hyperplasia, polyps, endometriosis, carcinoma, and less frequently mesenchymal and mixed epithelial-mesenchymal uterine tumors such as adenomyoma, leiomyoma, and adenosarcoma^(3)^. Endometrial adenocarcinoma is the most common malignancy associated with tamoxifen therapy. Both dosage and duration of treatment may be related to the development of uterine malignancies; tamoxifen dosage as low as 20 mg/day has been shown to increase cancer risk in patients. Moreover, tamoxifen treatment for longer than 2 years is accepted as the highest risk factor, although the majority of patients develop endometrial carcinoma within 2 years^(2)^. Tamoxifen-associated Mullerian adenosarcoma of the uterus has rarely been described. Thus, the estrogenic effect of tamoxifen on the endometrium may occasionaly also be complicated by mixed epithelial-mesenchymal or mesenchymal tumors. Endometrial stromal cells also contain estrogen receptors and Mullerian adenosarcomas are occasionally associated with long-term tamoxifen therapy^(2,3,4,5,6,7,8,9,10,11)^.

Mullerian adenosarcoma is differentiated from the more common uterine carcinosarcomas by the presence of a benign or atypical neoplastic glandular component with a well-differentiated epithelial lining and low-grade malignant sarcomatous stromal structure, and biologic behaviors that show low malignant potential^(1)^. The majority of patients who are diagnosed as having Mullerian adenosarcoma are postmenopausal and they present with abnormal vaginal bleeding or abdomino-pelvic pain. These tumors are usually single solitary polypoid masses that arise from the uterine fundus and project to the endometrial cavity, which appear to contain necrotic soft areas^(4,10)^. Treatment of primary disease is surgical with total abdominal hysterectomy but the role of staging with lymphadenectomy and/or oophorectomy is a subject of debate. A separate analysis of the Surveillance, Epidemiology, and End Results (SEER) database reported nodal metastases in 3.1% of 262 women who underwent lymphadenectomy for adenosarcoma. The reported experiences to date indicate that lymphadenectomy may not be necessary for staging all patients with uterine adenosarcoma. There is no consensus regarding complementary treatments. The benefit of such therapy in an adjuvant setting has not been demonstrated. Patients with sarcomatous overgrowth, deep myometrial invasion, lymphovasculer space invasion, and higher stage disease, which would indicate a worse prognosis, are more likely to have nodal metastases. Therefore, physicians consider adjuvant therapy regardless of nodal status. Most patients are diagnosed at an early stage and have a good prognosis with surgery alone but they have a higher risk of recurrence and death because of sarcomatous overgrowth and advanced stage disease^(12)^.

To our knowledge, only 16 cases of Mullerian adenosarcoma following tamoxifen therapy have been reported in the literature^(2,3,4,5,6,7,8,9,10,11)^. There are also three cases of uterine adenosarcoma in patients who had used hormonal agents for a previous diagnosis of breast cancer, reported by Carroll et al.^(12)^. In these 16 cases, the women had received tamoxifen for periods of 5 months or up to 4 years, but only 2 patients, who were reported by Jagavkar et al.^(5)^ and Arici et al.^(6)^ had received tamoxifen for more than 5 years. The patients’ age range in the literature is 42-76 years; the youngest patient was reported by Farhat and Fakhruddine,^(11)^ a woman agaed 42 years.

Our aim was to report the youngest woman in the literature with Mullerian adenosarcoma who was undergoing tamoxifen therapy for breast cancer. As in the literature, endometrial sampling was not performed before tamoxifen therapy. Therefore, it is difficult to understand the relation between tamoxifen and pathologic diagnosis. The role of tamoxifen therapy in the development of Mullerian adenosarcoma remains unclear. Mullerian adenosarcoma screening with endometrial biopsy is not necessary before and during tamoxifen therapy, but all cases of endometrial thickening and vaginal bleeding must be investigated for Mullerian adenosarcoma in tamoxifen users.
